# Cinnamaldehyde Suppressed EGF-Induced EMT Process and Inhibits Ovarian Cancer Progression Through PI3K/AKT Pathway

**DOI:** 10.3389/fphar.2022.779608

**Published:** 2022-05-12

**Authors:** Yue Wang, Ying Li, Liang Wang, Buze Chen, Miaolin Zhu, Chunyi Ma, Chunyan Mu, Aibin Tao, Shibao Li, Lan Luo, Ping Ma, Shuai Ji, Ting Lan

**Affiliations:** ^1^ Xuzhou Key Laboratory of Laboratory Diagnostics, Xuzhou Medical University, Xuzhou, China; ^2^ School of Medical Technology, Xuzhou Medical University, Xuzhou, China; ^3^ Department of Laboratory Medicine, Affiliated Hospital of Xuzhou Medical University, Xuzhou, China; ^4^ Department of Gynecology, Affiliated Hospital of Xuzhou Medical University, Xuzhou, China; ^5^ Department of Pathology, Jiangsu Cancer Hospital, Nanjing, China; ^6^ Division of Cardiology, Department of Medicine, The Affiliated People’s Hospital of Jiangsu University, Zhenjiang, China; ^7^ School of Pharmacology, Xuzhou Medical University, Xuzhou, China

**Keywords:** cinnamaldehyde, epithelial-to-mesenchymal transformation, Pi3k/AKt, ovarian cancer, proliferation, metastasis

## Abstract

Ovarian cancer is one of the most common gynecological malignancies in women worldwide with a poor survival rate. Cinnamaldehyde (CA), a bioactive substance isolated from cinnamon bark, is a natural drug and has shown that it can inhibit the progression of other tumors. However, the role of CA in ovarian cancer and its mechanism is poorly understood. In this study, wound healing assays, plate cloning, CCK-8, and transwell assays were used to determine cell proliferation and invasion. Western blot and flow cytometry were used to detect apoptosis levels. Western blot and immunofluorescence were used to detect changes in cellular EMT levels. The Western blot was used to detect levels of the PI3K/AKT signaling pathway. *In vivo*, we established a subcutaneous transplantation tumor model in nude mice to verify the role of CA in the progression and metastasis of ovarian cancer. Our data showed that *in vitro* CA was able to inhibit the cell viability of ovarian cancer. The results of scratch assay and transwell assay also showed that CA inhibited the proliferation and invasion ability of A2780 and SKOV3 cells. In addition, CA promoted apoptosis by increasing the expression of cleaved-PARP and cleaved-caspase 3 in ovarian cancer cells. Mechanistically, we found that CA inhibited the EGF-induced PI3K/AKT signaling pathway and reduced the phosphorylation levels of mTOR, PI3K, and AKT. The EGF-induced EMT process was also abolished by CA. The EMT process induced by AKT-specific activator SC79 was also suppressed by CA. Furthermore, in *in vivo*, CA significantly repressed the progression of ovarian cancer as well as liver metastasis. In all, our results suggest that CA inhibits ovarian cancer progression and metastasis *in vivo* and *in vitro* and inhibits EGF-induced EMT processes through the PI3K/AKT signaling pathway.

## Introduction

Ovarian cancer is one of the deadliest gynecological tumors, with approximately 239,000 diagnoses and 152,000 deaths worldwide each year (Reid et al., 2017; Kossaï et al., 2018; Zheng et al., 2019). Due to the lack of effective early screening for ovarian cancer, most patients are diagnosed at an advanced stage. The 5-year survival rate for patients diagnosed with stage III or IV ovarian cancer is less than 25%. Currently, first-line treatment for ovarian cancer mainly includes surgery and adjuvant chemotherapy. Although most patients are sensitive to therapeutic agents in the early stages of treatment, most of them eventually develop chemotherapy resistance (Odunsi, 2017). The main drugs used clinically for ovarian cancer treatment include platinum-based drugs, paclitaxel-based drugs, bevacizumab, and molecularly targeted drugs. Some studies reported that these drugs prolonged progression-free survival and 5-year survival, but there are still disadvantages such as more adverse reactions, drug resistance, broad-spectrum anti-cancer, and high prices (Chandra et al., 2019). Therefore, exploring efficient molecular-driven drugs to prevent the migration of ovarian cancer is urgently needed.

Epithelial–mesenchymal transition (EMT) is thought to be closely associated with tumor progression, including an increased ability to invade the extracellular matrix with metastasis to distant organs, resulting in a progressive transformation of cancer into a high-grade malignancy. During EMT, tumor cells lose their polarity and cellular adherence, which are characterized by the reduction of epithelial marker E-cadherin, and obtain motility and invasiveness, which are characterized by mesenchymal markers N-cadherin and vimentin. Transcriptional factors (TFs) are the primary clues in initiating EMT, such as Snail, TWIST, ZEB, and helix-loop-helix factors Twist1 and Twist2, which by binding to each other can regulate N-cadherin, E-cadherin, and vimentin expression, and also Snail, ZEB1, and ZEB2 are involved in the regulation of cell polarity-related protein expression to enhance the invasive ability of tumor cells (Batlle et al., 2000; Cano et al., 2000; Herranz et al., 2008). Additionally, transforming growth factor (TGF), epidermal growth factor (EGF), and hepatocyte growth factor (HGF) play an important role in the development of EMT and further promote the process of EMT and the ability of tumor proliferation and distant spread through the activation of downstream pathways, including PI3K/AKT/mTOR, MEK/ERK, WNT, and NOTCH pathways (Dongre and Weinberg, 2019). The PI3K/AKT signaling pathway is the most common signaling pathway that is aberrantly activated in ovarian cancer. Phosphatidylinositol 3-kinase (PI3K) is an upstream lipid kinase of the PI3K/AKT signaling pathway that can be activated by receptor tyrosine kinases (RTKs) and G protein-coupled receptors (GPCRs) to generate phosphatidylinositol (3,4,5)-trisphosphate (PIP3), and as a second messenger, PIP3 is able to recruit and activate the phosphorylation of downstream target proteins, amplify the action of PI3K, and maximize the activation of the AKT signaling pathway to exert pro-cell survival effects (Lien et al., 2017; Papa and Pandolfi, 2019).

Recently, there has been increasing interest in the development of chemotherapeutic reagents for cancer using natural products that are less toxic and have fewer unpredictable side effects than conventional synthetic drugs. Cinnamaldehyde (CA), a bioactive substance isolated from cinnamon bark, has been widely used as a natural flavor in food processing and related industries (Zhu et al., 2017). In recent years, the role of CA in inflammatory and bacterial, cardiovascular, and diabetes mellitus diseases have been known, and CA also played an anti-tumor role in various cancers (Elhennawy et al., 2021; Lee et al., 2018; Nour et al., 2018; Abdelmageed et al., 2019; Doyle and Stephens, 2019; Ying et al., 2019; Wang et al., 2020). For example, CA acts in synergy with oxaliplatin to reverse nodal hypoxia-induced EMT by inhibiting the Wnt/β-catenin pathway and exerting anti-tumor effects in colorectal cancer (Wu et al., 2019). Liu et al. (2020) identified the antioxidant effects of CA in breast cancer and could inhibit cell growth and promote apoptosis through PI3K/AKT and NF-κB pathways. However, the role of CA in the treatment of ovarian cancer is still unclear.

In this study, we aim to investigate the effect of the natural drug CA on ovarian cancer cell progression, to determine whether CA inhibited the EGF-induced EMT progress in ovarian cancer A2780 and SKOV3 cell lines, and to further clarify the molecular mechanism of CA on ovarian cancer cells to explore a new effective natural anti-tumor drug for ovarian cancer treatment.

## Materials and Methods

### Reagents and Antibodies

Cinnamaldehyde (Aladdin, Shanghai, China) has the chemical formula C_9_H_8_O, a relative molecular mass of 132.16, and a purity of >99.5%. EGF was purchased from MedChemExpress (MCE, Shanghai, China).

Antibodies against mTOR, p-mTOR (Ser2448), pan-AKT, p-AKT (Ser473), PI3K, p-PI3K(Tyr458), E-cadherin, N-cadherin, vimentin, and Snail were purchased from Affinity Biosciences (Affinity Biosciences, OH, United States). Antibodies against Ki67, MMP9, EGFR (Tyr1068), cleaved-PARP, PARP, cleaved-caspase-3, and caspase-3 were purchased from Cell Signaling Technology (Danvers, MA, United States). The antibody against β-actin was purchased from Proteintech (Proteintech, Chicago, United States).

### Cell Lines

Human ovarian cancer cell lines SKOV3 and A2780 and the human normal epithelial cell line IOSE80 were obtained from the American Type Culture Collection (ATCC). IOSE80 cell lines were cultured in Roswell Park Memorial Institute-1640 (RPMI-1640) medium (Kaiji, Jiangsu, China) with 10% fetal bovine serum (FBS) and 1% penicillin–streptomycin (P/S, Invitrogen). Ovarian cancer cells were cultured in Dulbecco’s modified Eagle’s medium (DMEM) (Kaiji, Jiangsu, China) containing 10% fetal bovine serum (Gibco, Grand Island, NY, United States). Cell culture was performed at 37°C with 5% CO_2_ in the air. When cell fusion reached 70–80%, EMT was induced by treating 100 ng/ml EGF with the DMEM. Meanwhile, EMT was administered at appropriate concentrations of CA for 48 h.

### CCK8

Human A2780, SKOV3, and IOSE80 cells were placed in 96-well plates, and CA was added accordingly to different groups and incubated for 24, 48, or 72 h, and CCK-8 reagent (Kaiji, Jiangsu, China) was added under light-proof conditions, and the absorbance was measured at 450 nm after 2 h in the incubator. The calculation formula of cell viability is as follows: cell viability (%) = [A (CA)-A (blank)]/[A (0 µg/ml CA)-A (blank)] × 100%, where A (CA) is absorbance of a well with cells, CCK-8 solution, and CA; A (blank) is absorbance of a well with medium and CCK-8 solution, without cells; A (0 µg/ml CA) is absorbance of a well with cells and CCK-8 solution, without CA solution.

### Colony Formation

The cells were placed in 6-well plates at 1 × 10^3^ cells/well, and the cells were plastered and added with the 0.5, 1, 5, and 10 ug/ml of CA according to the grouping, incubated for 2 weeks, washed three times with phosphate-buffered saline (PBS), fixed with 4% paraformaldehyde, and stained with 0.05% crystal violet. Then, the six-well plates were gently washed with water and air-dried at room temperature. Photographs were taken under a microscope and measured with ImageJ software.

### Wound Healing

The A2780 and SKOV3 cells were cultured in monolayers in six-well plates and grown at 90% fusion. Artificial wounds were gently created with a 10-μl pipette tip. After washing with PBS to remove isolated cells, CA-cultured cells were added to the DMEM medium containing 10% fetal bovine serum in different groups. Micrographs were taken at 0, 24, and 48 h after CA treatment to observe the cell migration distances. Scratch distances were measured and calculated as migration rates.

### Transwell

The difference between the transwell migration and invasion assay methods is that the invasion assay requires pre-coating of the basement membrane in the upper layer of the cell, and in addition, the incubation time of the invasion assay is 24 h longer than that of the migration assay. The A2780 and SKOV3 cells were inoculated with serum-free DMEM dilution in the upper layer of the cell, and CA was dissolved in DMEM according to different groups and added to the lower layer of the cell and incubated for 24 or 48 h. After incubation, the cells on the upper surface of the insert were wiped with cotton swabs, and the migrating cells were fixed with 4% paraformaldehyde and stained with 0.05% crystal violet. The migrated cells were photographed under an inverted fluorescence microscope, and five random fields were selected for quantification.

### Western Blotting

To confirm the expression levels of intracellular proteins, Western blotting was performed using EGF CA treated cells for the appropriate time. After incubation, cells were washed with cold PBS. The cells were harvested with a spatula, centrifuged, and the resulting precipitate was lysed in RIPA buffer at 4°C for 10 min, vortex shaken for 1 min, and repeated three times. Supernatants containing intracellular proteins were obtained by centrifugation at 12,000 g for 30 min. Protein concentration was determined by the BCA Protein Assay Kit (Kaiji, Jiangsu, China) and quantified. The SDS-PAGE gel separation was performed. The proteins were transferred to polyvinylidene difluoride (PVDF) membranes and incubated with primary antibody overnight at 4°C. After incubation with species-specific horseradish peroxidase (HRP)-labeled secondary antibodies, immunoreactive proteins were visualized by using an ECL reagent (Yeasen Biotechnology, China) in Image Lab Software (Bio-Rad, United States).

### Cell Apoptosis

The Annexin V/APC staining kit (Kaiji, Jiangsu, China) was used to analyze apoptosis. Briefly, 1 × 10^5^ cells were suspended in 100 µl buffer and 5 µl FITC Annexin V, and 5 µl APC was added and incubated for 15 min at room temperature. After incubation, cells were analyzed by flow cytometry.

### Immunofluorescence Staining

The CA-treated A2780 and SKOV3 cells for 48 h were fixed in 4% paraformaldehyde and then incubated with 0.1% Triton X-100. Subsequently, the cells were blocked with 5% BSA and incubated overnight at 4°C with primary antibodies against E-cadherin and N-cadherin. The cells were then incubated with Alexa Flour donkey anti-rabbit secondary antibody for 2 h and stained with DAPI for 10 min. Finally, the fluorescent signal was observed and detected under inversion.

### Tumor Xenograft

For the animal model, six-week-old female nude mice were purchased from the Xuzhou Medical University Experimental Animal Center. The A2780 cells (5 × 10^6^) were resuspended with 200 µl 1% matrige (Corning, New York, United States) and injected into the peritoneal cavity of nude mice. In terms of drug therapy, mice in the control group were intraperitoneally injected with 100 µl phosphate buffer saline, and mice in the low-dose and high-dose groups were intraperitoneally injected with 50 mg/kg and 100 mg/kg CA every 3 days, respectively. Meanwhile, the bodyweight of the nude mice was weighed and recorded. After three weeks, the mice were killed for an autopsy, and the tumors were weighed. Immunohistochemistry and HE staining were performed on the tumor tissues, liver, and lung tissues. All methods were performed according to the guidelines approved by the Ethics Committee of the Experimental Animal Research Center of Xuzhou Medical University.

### Immunohistochemistry

The tumor tissue from nude mice was dewaxed and rehydrated and incubated in 3% H_2_O_2_ for 15 min at room temperature and protected from light to block peroxidase. Antigen repair was performed in citric acid antigen repair buffer (PH = 6) followed by blocking with 10% BSA and incubated with antibodies against Ki67, MMP9, or EGFR (Affinity, Shanghai, China) overnight at 4°C, followed by the secondary antibody for 50 min at room temperature. Drops of chromogenic solution were added, hematoxylin was restrained, and the slices were dehydrated and blocked. The images were obtained using an Olympus microscope and the image analysis was performed.

### Statistics

Each experiment was performed at least three times. Data were shown as mean ± standard deviation (SD) and analyzed by SPSS software (version 19.0). The statistical comparison between groups was conducted *via* Student’s t-tests or one-way analysis of variance (ANOVA). *p* < 0.05 was considered statistically significant.

## Results

### Effect of Cinnamaldehyde on Cell Proliferation in A2780 and SKOV3 Ovarian Epithelial Cancer Cells as Well as IOSE80 Normal Ovarian Epithelial Cell

**FIGURE 1:**
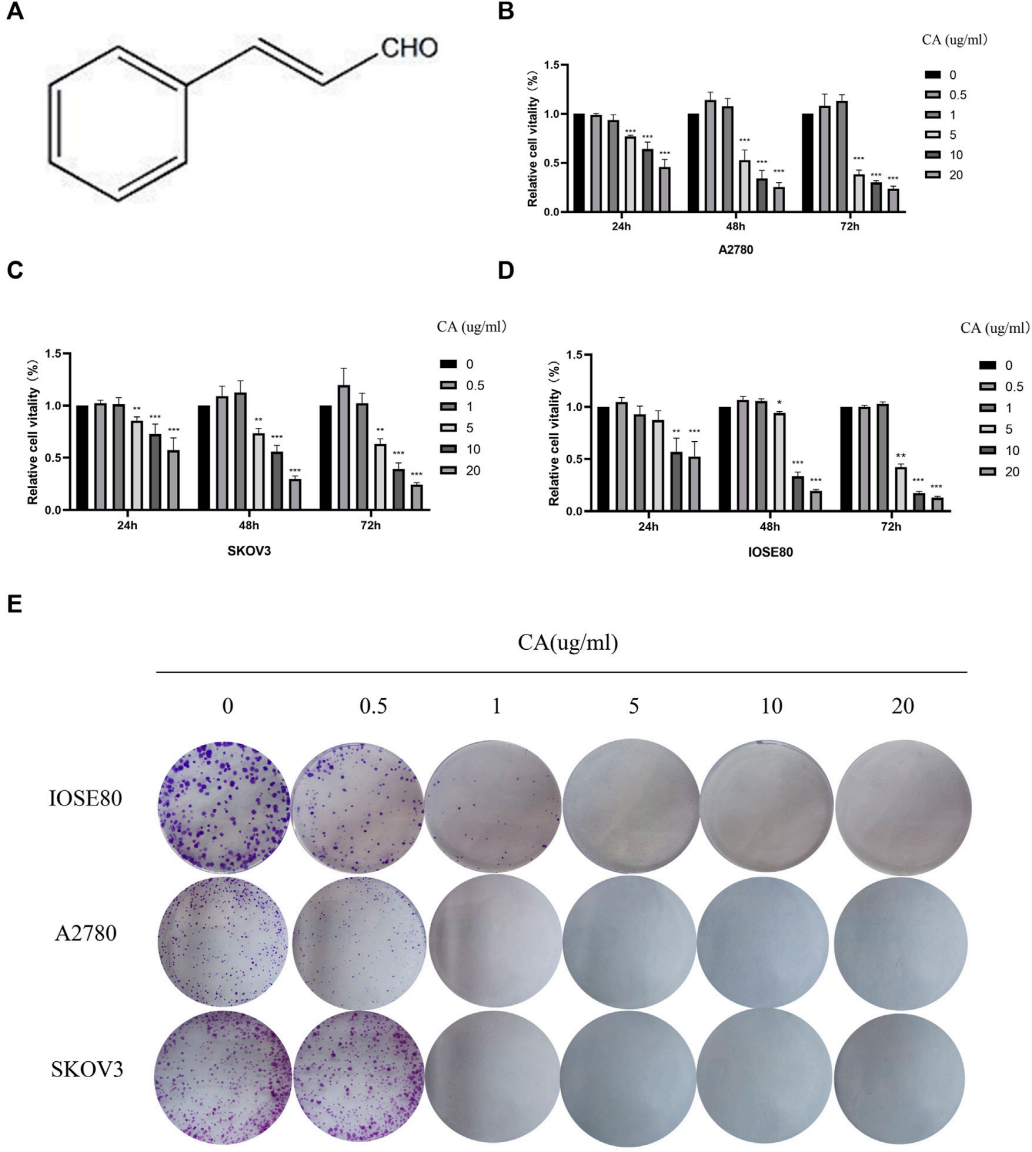
CA inhibited the proliferation of A2780 and SKOV3 cells *in vitro*. **(A)** Molecular structure of CA. CA-treated A2780 **(B)**, SKOV3 **(C)**, and IOSE80 **(D)** cells for 24, 48, 72 h, and cell viability was detected by CCK-8. **(E)** Ability of cells to proliferate was assayed with the colony formation assay. The experimental results were quantified using ImageJ software, mean ± SD * *p* < 0.05, ***p < 0.001. The experiments were performed independently three times.

First, in order to explore the effect of CA on the proliferation in A2780, SKOV3, and IOSE80 cells, CCK-8 assay and colony formation assay were conducted. As shown in Figures 1B, C, the proliferation of A2780 and SKOV3 cells decreased in the dose-dependent and time-dependent manner upon treatment with CA at a concentration of above 5 ug/ml for 24, 48, or 72 h. However, CA had lower effect on the proliferation of normal ovarian epithelial cells IOSE80 than A2780 and SKOV3 cells; there was significant inhibition in cell proliferation at a concentration of more than 10 ug/ml (Figure 1D). Therefore, we chose 0.5, 1, 5, and 10 ug/ml of CA for the follow-up experiments. In addition, the IC_50_ values of IOSE80, A2780, and SKOV3 cells after 24 h of CA treatment were 19.18 ug/ml, 11.82 ug/ml, and 23.00 ug/ml, respectively. The IC_50_ values of IOSE80, A2780, and SKOV3 cells after 48 h of CA treatment were 5.956 ug/ml, 8.651 ug/ml, and 5.792 ug/ml, respectively. The IC_50_ values of IOSE80, A2780, and SKOV3 cells after 72 h of CA treatment were 15.80 ug/ml, 7.059 ug/ml, and 7.096 ug/ml, respectively. Meanwhile, plate cloning experiments showed that CA could inhibit the colony-forming ability of ovarian epithelial cancer cells and normal ovarian epithelial cells (Figure 1E). Taken together, both CCK-8 and plate cloning assays demonstrated that CA inhibited the proliferation capacity of epithelial ovarian cancer cells in a concentration gradient.

### Effect of Cinnamaldehyde on Cell Migration, Invasion, and Apoptosis of Epithelial Ovarian Cancer Cells

**FIGURE 2:**
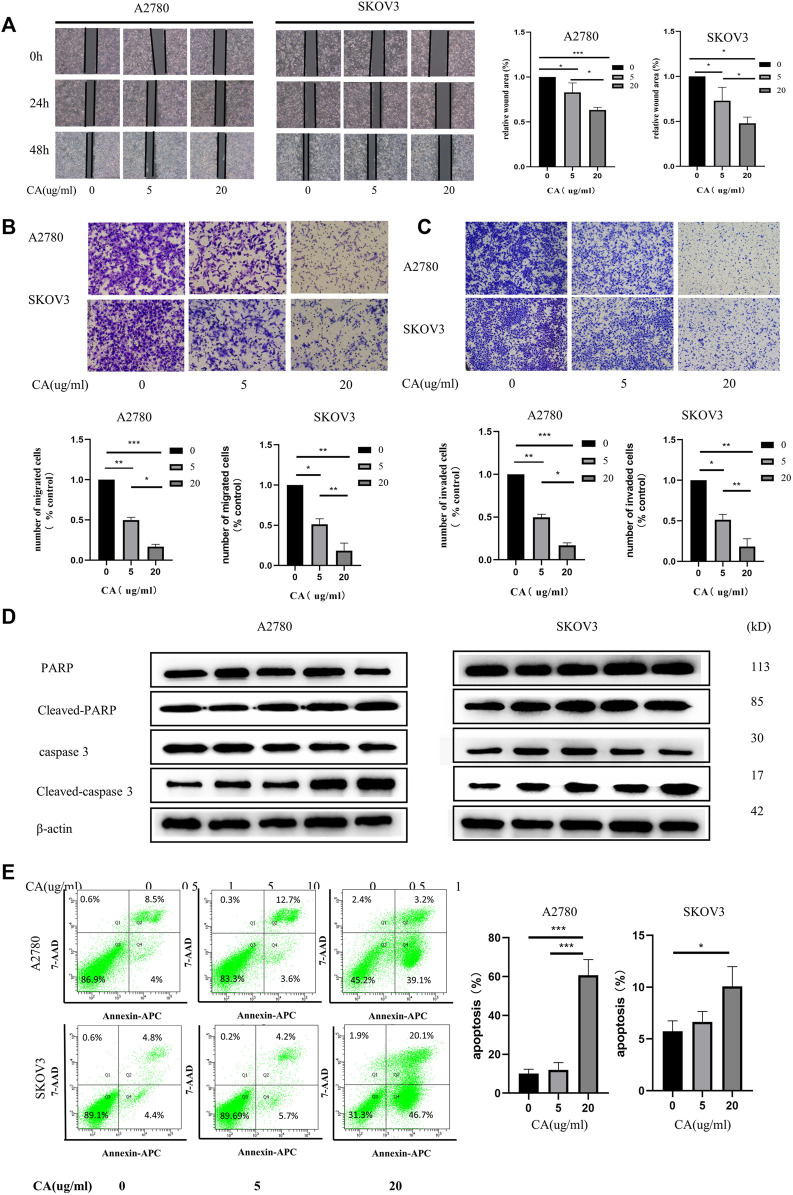
CA induced apoptosis and inhibited the metastatic biological functions of A2780 and SKOV3 cells *in vitro*. **(A)** Images of wound healing assays of SKOV3 and A2780 cells co-cultured with CA for 0, 24, and 48 h as well as relative quantitative data of cell trauma. The transwell assay was used to detect the effect of CA on the migratory **(B)** and invasive **(C)** abilities of SKOV3 and A2780 cells that are shown as representative plots and quantitative data of cells, respectively. **(D)** Western blotting detected the expression of apoptosis-related proteins after CA treatment of A2780 and SKOV3. **(E)** Flow cytometry determined the apoptosis levels of A2780 and SKOV3 cells after CA treatment. Representative images are shown separately. Results are expressed as mean ± SD (*n* = 3). *p < 0.05, *p < 0.01, ***p < 0.001.

Next, we used wound the healing and transwell assays to investigate the effect of CA on the migration and invasion ability of ovarian cancer cells *in vitro*. As shown in Figure 2A, CA significantly inhibited the speed of wound healing of ovarian cancer cells in a concentration-dependent manner. Consistently, the transwell assay found that the numbers of SKOV3 and A2780 cells were suppressed by the treatment of CA (Figure 2B). Furthermore, to measure whether CA suppresses the invasion of A2780 and SKOV3 cells, a Matrigel transwell invasion assay was employed. Similarly, the invasive cells of the CA group decreased in a dose-dependent manner which was compared to the control group (Figure 2C). The wound healing assay and transwell assay both suggested that CA could inhibit the migration and invasion ability of ovarian cancer cells.

Flow cytometry was used to analyze the function of CA on the apoptosis of ovarian cancer cells. The results showed that the apoptosis rate of ovarian cancer cells was significantly increased after CA treatment, especially at 20 ug/ml (Figure 2D). Following this, the expression of cleaved-caspase-3 and cleaved-PARP were detected using Western blotting. Caspase-3 is a critical executioner of apoptosis, as it is either partially or totally responsible for the proteolytic cleavage of many key proteins, such as the nuclear enzyme poly (ADP-ribose) polymerase (PARP). PARP is one of the major apoptotic markers and is responsible for repairing stress or DNA damage. PARP is converted to cleaved-PARP, a classical substrate for caspase 3, which leads to cell lysis or death (Wang et al., 2018; Yadav et al., 2021). As shown in Figure 2E, CA upregulated the expression of cleaved-PARP and cleaved-caspase. Together these results imply that CA not only inhibited the migration and invasion in a concentration-dependent manner but also promoted the apoptosis of ovarian epithelial cancer cells through the caspase3 and PARP pathways.

### Cinnamaldehyde Suppresses EGF-Induced EMT in Human Epithelial Ovarian Cancer Cells

**FIGURE 3:**
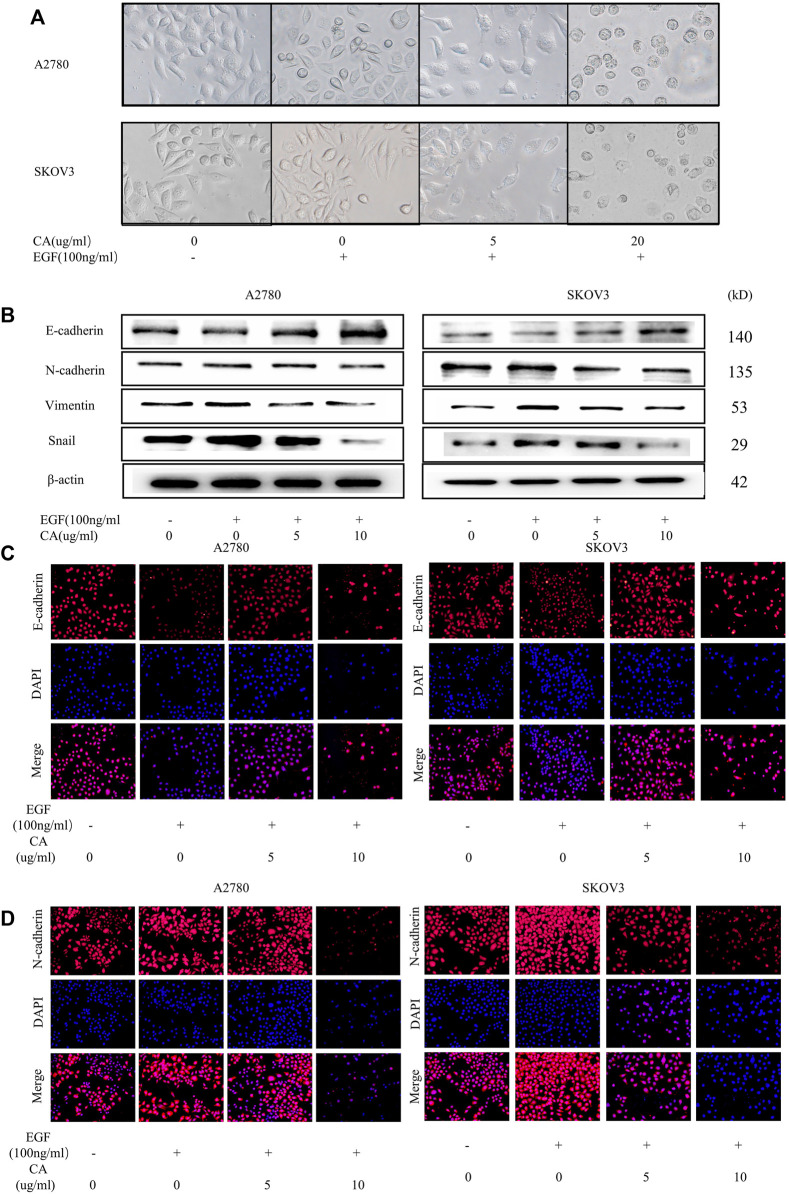
CA reverses the EGF-induced EMT process in A2780 and SKOV3 cells *in vitro*. **(A)** Cell morphology of A2780 and SKOV3 after CA and EGF treatment. **(B)** Expression of E-cadherin, N-cadherin, vimentin, and Snail were detected by Western blotting in A2780 and SKOV3 cells after CA and EGF treatment. Representative fluorescence images of E-cadherin **(C)** and N-cadherin **(D)** in A2780 and SKOV3 cells. At least three independent experiments were performed.

To clarify the effect of CA on EMT in epithelial ovarian cancer cells by EGF, A2780 and SKOV3 cells were treated with only EGF (100 ng/ml) or EGF plus CA (5 or 10 ug/ml) for 48 h. As shown in Figure 3A, the A2780 and SKOV3 cells stimulated with EGF showed a scattered distribution and elongated spindle-shaped mesenchymal appearance compared with the control group, and then the epithelial characteristics of the cells were gradually enhanced by adding CA. The main biomarkers of EMT including E-cadherin, N-cadherin, vimentin, and Snail were detected. In our study, E-cadherin, an epithelial cell marker, was decreased after EGF stimulation and increased after CA treatment. However, the mesenchymal cell markers, N-cadherin, vimentin, and Snail, were increased after EGF stimulation and then decreased with the different CA concentrations (Figures 3B, C). Similarly, immunofluorescence results indicated that EGF decreased the expression of E-cadherin and increased the expression of N-cadherin in the cells compared with the control group, and these changes were reversed after CA treatment (Figures 3D–G). All the results indicated that CA inhibited epithelial ovarian cancer cells migration and invasion by reversing EMT.

### Cinnamaldehyde Suppressed EMT in Human Epithelial Ovarian Cancer Cells by PI3K/AKT Signaling Pathway

**FIGURE 4:**
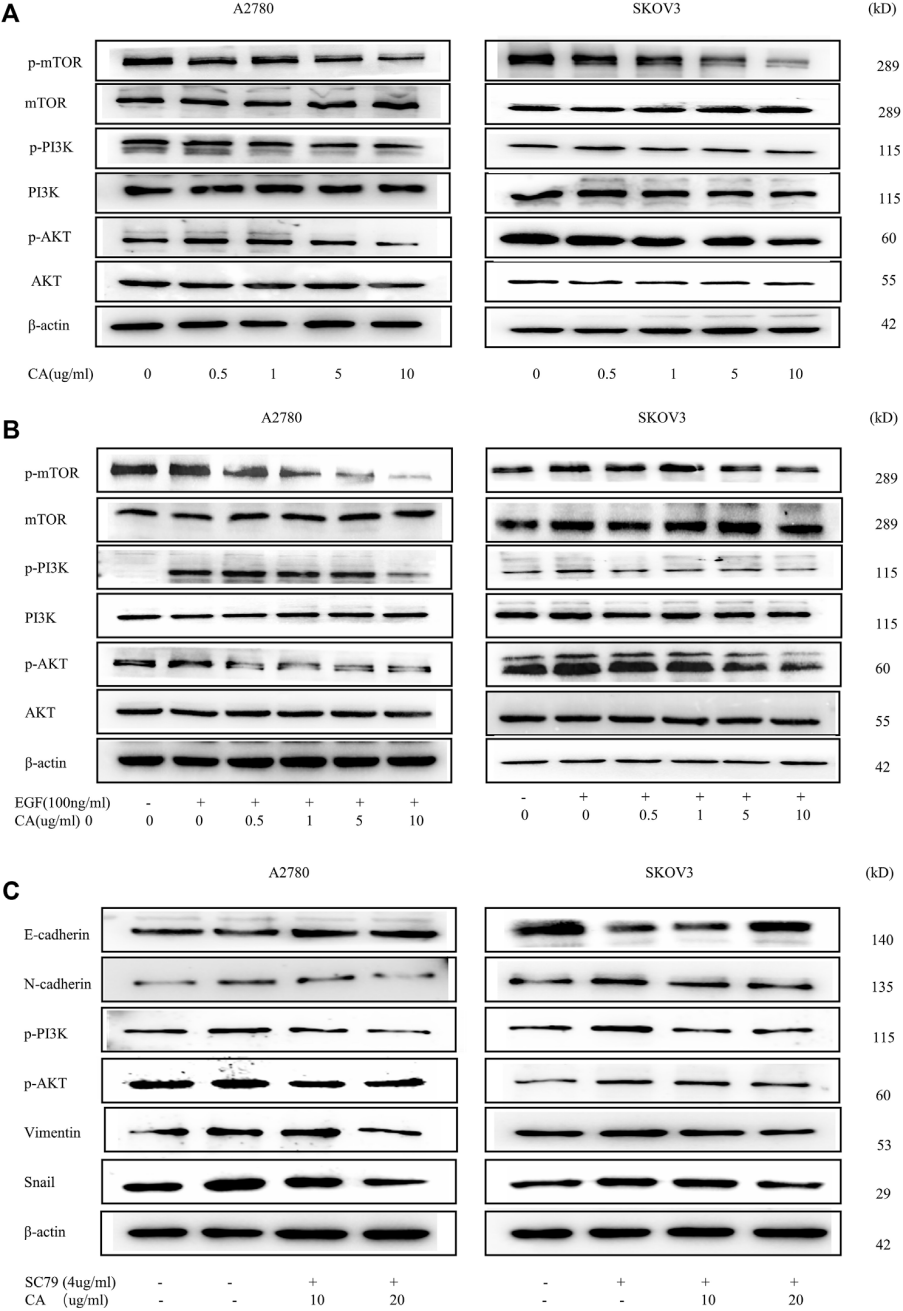
CA reverses the EMT process of A2780 and SKOV3 *via* the PI3K/AKT signaling pathway. **(A)** Phosphorylation levels of mTOR, PI3K, and AKT were detected by Western blotting in A2780 and SKOV3 cells. **(B)** Phosphorylation levels of mTOR, PI3K, and AKT were detected by Western blotting in A2780 and SKOV3 cells after CA and with or without EGF treatment. **(C)** Expression levels of E-cadherin, N-cadherin, Snail, and vimentin were measured by Western blotting in A2780 and SKOV3 cells. Representative images are shown separately.

EMT has already been shown to be regulated by multiple signaling pathways, including the PI3K/AKT signaling pathway. To clarify the effect of CA on the PI3K/AKT signaling pathway, we examined the phosphorylation expression levels of the major proteins of the signaling pathway using Western blotting. Results showed that CA concentration-dependent inhibited the PI3K/AKT signaling molecules including phosphorylated AKT, PI3K, and mTOR (shown in Figures 4A, B). Meanwhile, we found that the PI3K/AKT signaling pathway was activated upon EGF stimulation, and CA reversed the facilitation of EGF with a concentration gradient (Figures 4C, D). To further investigate whether CA reversed the EMT process through the PI3K/AKT signaling pathway, we used SC79, the AKT specific activator, to treat A2780 and SKOV3 cells. As shown in Figures 4E, F, the SC79 promoted the EMT process in ovarian cancer cells. However, after the addition of CA, the expression levels of N-cadherin, vimentin, and Snail gradually downregulated and the expression level of E-cadherin upregulated. These results revealed that CA can reverse the EGF-mediated EMT process *via* the PI3K/AKT signaling pathway in epithelial ovarian cancer cells.

### Cinnamaldehyde Inhibits the Growth of Epithelial Ovarian Cancer Cells *In Vivo*

**FIGURE 5:**
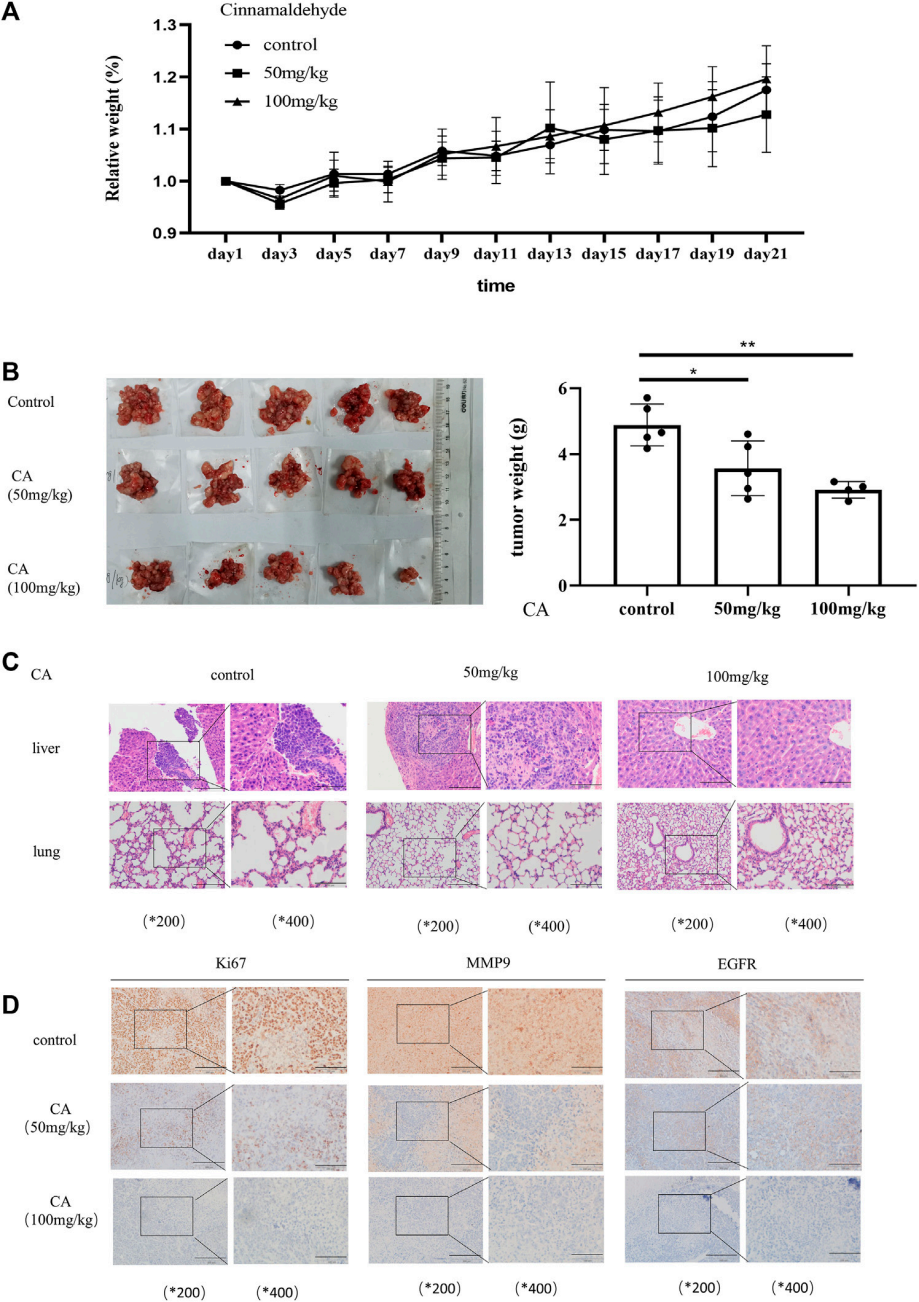
CA suppresses ovarian tumor growth *in vivo*. **(A)** Bodyweight changes in nude mice injected with A2780 cells and administered with PBS or CA (50 or 100 mg/kg). **(B)** Quantitative data of tumor weight in PBS or CA (50, 100 mg/kg)-administered nude mice. **(C)** Representative images of liver and lung metastases in nude mice after injection of A2780 cells and administration of PBS or CA (50 or 100 mg/kg). **(D)** Expression of EGFR, Ki67, and MMP9 in tumor tissues of nude mice. Results are presented as mean ± SD (*n* = 5). *p ≤ 0.05; **p ≤ 0.01.

In order to confirm whether CA can play the same role in *in vivo* as *in* vitro, we established the subcutaneous xenograft model of the A2780 cells in nude mice. In the xenotransplantation model, mice were monitored and weighed every 3 days and killed at the third week. As shown in Figure 5A, the weight of mice was a steady raising trend, and there was no statistical significance between the three groups, indicating that CA was harmless to the mice. Meanwhile, the weight of abdominal tumor in mice decreased in the CA groups, especially in 100 mg/kg groups (Figures 5B, C). HE staining of paraffin sections of the liver and lung tissue of the mice showed that three of five mice in the control group developed liver metastasis of ovarian cancer, but one of five mice in the low-dose CA group showed liver metastasis, and even the group treated with CA of 100 ug/kg showed no mouse with liver metastasis (Figure 5D). Moreover, the three groups showed no lung metastasis (Figure 5E). Immunohistochemical results of the tumor tissue show that the expression levels of Ki67 and MMP9 as well as EGFR were significantly reduced in CA-treated groups compared with the CA-untreated group (Figure 5F). Consistent with the aforementioned results, CA significantly inhibits the growth and metastasis of ovarian cancer cells *in vivo*.

## Discussion

Ovarian cancer is still considered a big problem for female health despite treatment progress in recent years. Consequently, explorations for an agent effectively suppressing ovarian cancer cell proliferation and invasion may provide novel therapeutic options. In this study, we aimed to study the anti-tumor activity of CA in ovarian cancer and explored the underlying mechanisms. Our results suggested that CA inhibited ovarian cancer cell migration and invasion by reversing EMT through the PI3K/AKT signaling pathway for the first time.

In recent years, several studies have been conducted on CA, especially in anti-inflammatory and therapeutic cardiovascular diseases CA has shown good performance. CA has been reported to be able to reduce the inflammatory response in ulcerative colitis by modulating the JAK2/STAT3 pathway (Elhennawy et al., 2021). Nour et al. (2018), Abdelmageed et al. (2019), and Wang et al. (2020) also found that CA not only reduces atherosclerosis induced by high-cholesterol diet in rabbits but also has therapeutic potential for diabetes mellitus. A number of studies have identified that CA inhibited the proliferation and metastasis of cancers (Li et al., 2016; Wu et al., 2019; Liu et al., 2020). Liu et al. (2020) reported that CA inhibited the proliferation and invasion of breast cancer, and the PI3K/AKT signaling pathway is its potential target through KEGG analysis. Li et al. (2016) found that CA reduced the expression level of MMPs, the key proteins that downregulated cell adhesion ability in colorectal cancer cells. The improvement of cell adhesion ability is exactly what an indispensable key link for its invasion and metastasis (Li et al., 2016). Han et al. (2020) found that CA plays a tumor-suppressive role by inhibiting the growth of cancer-associated fibroblasts (CAFs) in prostate cancer. In our study, we found that treatment with more than 10 ug/ml CA significantly reduced the proliferation, migration, and invasive capacities of ovarian cancer cells (Figures 1, 2). This suggested that CA inhibited the proliferation, invasion, and migration ability of the A2780 and SKOV3 cells *in vitro*.

Apoptosis is a form of programmed cell death and an important component in maintaining tissue homeostasis (Kerr et al., 1972). Caspases exist as inactive zymogens and are involved in apoptosis *via* the death receptor pathway (Pore et al., 2013). During apoptosis, initiator caspases (e.g., caspases-8 and -9) are activated first, followed by effectors caspases (e.g., caspases -3 and -7) which are cleaved and activated by the promoter and cleave structural proteins, which in turn lead to cell death (Yadav et al., 2021). The DNA repair enzyme poly (ADP-ribose) polymerase (PARP) is one of the classical substrates of caspase-3 and can detect DNA strand breaks. Caspase-3 and PARP, especially cleaved-caspase-3 and cleaved-PARP, are key factors involved in the regulation of apoptosis (Wang et al., 2018). Several studies have found that CA plays a role in promoting cell apoptosis. Wu et al. (2019) found that CA promoted cell apoptosis *in vivo* and *in vitro* and enhanced the oxaliplatin sensitivity of tumor cells. We explored the proapoptotic effect of CA on A2780 and SKOV3 cells which is consistent with those of Wu et al. (2017) found in non-small cell lung cancer cells. In our study, flow cytometry results showed that CA was able to promote apoptosis levels in ovarian cancer cells, and Western blotting showed that CA was able to activate the cascade reaction of caspases and promote the expression of cleaved-caspase-3 and cleaved-PARP, demonstrating that CA was able to promote apoptosis by activating the caspase cascade reaction in ovarian cancer.

The process of metastasis and progression of malignant tumors is a complex process regulated by multiple mechanisms, including the reduction of tumor cell adhesion and alteration of the tumor microenvironment. When ovarian cancer metastasizes through the body cavity, the interconnection between tumor cells is weakened, which in turn detaches from the ovarian surface and colonizes other locations such as the greater omentum by the action of ascites (Yousefi et al., 2020). Mesothelial cells and mesenchymal stem cells, etc., contained in ascites and cytokines such as interleukins and chemokines secreted by the associated immune cells, blood, and nerves, etc., together constitute a microenvironment conducive to tumor cell metastasis and colonization. E-cadherin, an important marker for EMT, decreases in expression during tumor spread, and it is a key feature of EMT, which decreases the intercellular adhesion, decreases cell polarity, and metastasizes tumor cells easily with body fluids. N-cadherin and vimentin increase the mesenchymal phenotype of tumor cells and the invasiveness of cells. Snail, a transcription factor, can regulate the expression of E-cadherin and other transcription factors, such as ZEB-1 and ZEB-2, and promote EMT. Snail and vimentin are also thought to be associated with increased tumor aggressiveness (Dai et al., 2020). Therefore, inhibition of the EMT process in tumor cells is an important means of inhibiting tumor metastasis. EGFR is highly expressed in ovarian epithelial carcinoma and is thought to be closely associated with the EMT process (Sun et al., 2021). Previous studies have shown that CA can inhibit tumor cell growth by reversing the EMT process in non-small cell lung cancer (Wu et al., 2017), and CA combined with oxaliplatin can reverse hypoxia-induced EMT in colorectal cancer by collaborating (Wu et al., 2019). In our study, we explored the role of CA in the regulation of EMT in ovarian cancer, and we used the addition of EGF in the A2780 and SKOV3 cells to mimic the condition of the organism suffering from ovarian cancer. In our experiments, when the cells were treated with EGF, the EMT process was activated, and the cells were scattered with a long spindle-shaped mesenchymal feature. At the same time, the E-cadherin expression level was decreased and the N-cadherin expression level was increased, which is one of the important features of the EMT process. When CA stimulation was given again, the cells gradually assumed epithelial morphology, and Western blotting and immunofluorescence results showed that E-cadherin expression was significantly increased and N-cadherin, Snail, and vimentin expression levels were significantly decreased. These results indicate that CA can effectively inhibit the EMT process in the A2780 and SKOV3 cells.

In the development of ovarian cancer, several signaling pathways are aberrantly activated, including the PI3K/AKT signaling pathway, MEK/ERK signaling pathway, and Wnt/β-catenin signaling pathway, which are also coincidentally closely related to the EMT process (Dongre and Weinberg, 2019). Among them, the PI3K/AKT signaling pathway is the most frequently activated pathway (Ediriweera et al., 2019; Zhang et al., 2019; Nguyen et al., 2019), which is also involved in regulating cell proliferation, apoptosis, invasion, survival, and angiogenesis (Shao et al., 2020). Liu et al. (2020) used GeneCards and OMIM databases for the enrichment analysis and found that CA is closely related to the AKT/PI3K signaling pathway in breast cancer. Liu and Huang confirmed that CA regulated colorectal cancer and osteosarcoma progressing *via* the AKT/PI3K signaling pathway (Li et al., 2016; Huang et al., 2020). In our present study, Western blotting results showed that CA was able to downregulate the phosphorylation levels of PI3K/AKT pathway proteins in A2780 and SKOV3 cells with or without EGF. The PI3K/AKT signaling pathway was activated when A2780 and SKOV3 cells were stimulated with EGF, which has been shown to increase tumor proliferation and invasion. When given a certain concentration of CA stimulation, the phosphorylation levels of AKT, PI3K, and mTOR were reduced in a concentration-dependent manner. To further clarify that CA reverses the EMT process through the PI3K/AKT signaling pathway, we used SC79, a specific activator of AKT. The result showed that SC79 activated the EMT process and it was significantly inhibited after the addition of 20 ug/ml CA. These suggested that CA inhibited the EMT process by mediating the PI3K/AKT signaling pathway.

In the present study, we have validated the antimetastatic activity of CA *in vitro*. We constructed an ovarian cancer xenograft model by intraperitoneally growing A2780 cells in nude mice supporting that CA inhibited ovarian cancer metastasis *in vivo*. First, the results have shown that CA significantly reduced tumor volume, tumor weight, and tumor cell count *in vivo* as compared to control groups, which is similar with Huang et al. (2020) conclusion in osteosarcoma. Furthermore, to explore CA on the inhibition of ovarian cancer distant metastasis, which was regularly treated with CA and continuously tested for physical status (Figure 5). Our results showed that CA was able to inhibit the growth of abdominal tumors in the nude mice. In our study, the HE staining analysis is to observe liver and lung metastases. No lung metastasis was observed in both control and CA groups which is consistent with the results of the clinical study. But, different from lung metastasis, there is a lot of live metastasis in the control group, and CA significantly inhibited live metastasis, especially with the 20 mg/kg concentration. MMP9 has been found to be closely associated with metastasis in ovarian cancer and to act as an independent prognostic marker (Rasool et al., 2016; Yousefi et al., 2020). Ki67 is a well-known proliferation-associated protein that is highly expressed in cancer cells and almost rarely in normal cells, and Ki67 has emerged as an independent prognostic factor in certain cancers. As mentioned in our results, immunohistochemical results showed that CA significantly reduced the expression levels of MMP9 and Ki67 in tumor tissues (Yang et al., 2018). All in all, CA suppressed ovarian cancer growth and metastasis *in vivo*.

## Conclusion

In summary, our study reported the inhibitory effect of CA on the proliferation and invasion, growth, and metastasis of human epithelial ovarian cancer cells *in vitro* and *in vivo*, and this effect may be related to CA reversing the EMT process and inhibiting the proliferation and migration of ovarian cancer cells by downregulating the PI3K/AKT signaling pathway. This study suggests that CA is promising as a potential natural drug for the treatment of ovarian epithelial cancer.

## Data Availability

The original contributions presented in the study are included in the article/Supplementary Material, further inquiries can be directed to the corresponding authors.
