# Corrigendum: Cinnamaldehyde suppressed EGF-induced EMT process and inhibits ovarian cancer progression through PI3K/AKT pathway

**DOI:** 10.3389/fphar.2024.1427330

**Published:** 2024-07-08

**Authors:** Yue Wang, Ying Li, Liang Wang, Buze Chen, Miaolin Zhu, Chunyi Ma, Chunyan Mu, Aibin Tao, Shibao Li, Lan Luo, Ping Ma, Shuai Ji, Ting Lan

**Affiliations:** ^1^ Xuzhou Key Laboratory of Laboratory Diagnostics, Xuzhou Medical University, Xuzhou, China; ^2^ School of Medical Technology, Xuzhou Medical University, Xuzhou, China; ^3^ Department of Laboratory Medicine, Affiliated Hospital of Xuzhou Medical University, Xuzhou, China; ^4^ Department of Gynecology, Affiliated Hospital of Xuzhou Medical University, Xuzhou, China; ^5^ Department of Pathology, Jiangsu Cancer Hospital, Nanjing, China; ^6^ Division of Cardiology, Department of Medicine, The Affiliated People’s Hospital of Jiangsu University, Zhenjiang, China; ^7^ School of Pharmacy, Xuzhou Medical University, Xuzhou, China

**Keywords:** cinnamaldehyde, epithelial-to-mesenchymal transformation, PI3K/AKT, ovarian cancer, proliferation, metastasis

In the published article, there was an error in Figure 3 as published. The 5 μg/mL CA DAPI and the 10 μg/mL CA Merge got mixed up in Figure 3D. The corrected Figure 3 appear below.

**FIGURE 3:**
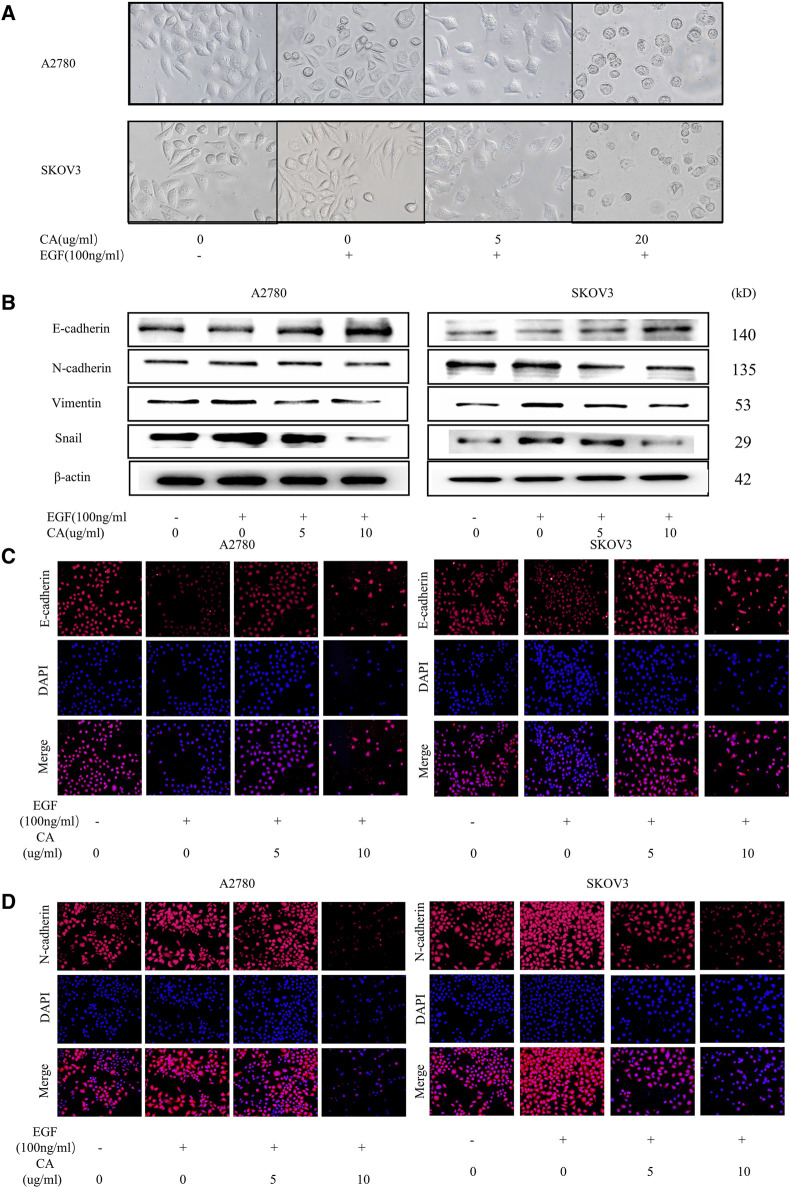
CA reverses the EGF-induced EMT process in A2780 and SKOV3 cells in vitro. **(A)** Cell morphology of A2780 and SKOV3 after CA and EGF treatment. **(B)** Expression of E-cadherin, N-cadherin, vimentin, and Snail were detected by Western blotting in A2780 and SKOV3 cells after CA and EGF treatment. Representative fluorescence images of E-cadherin **(C)** and N-cadherin **(D)** in A2780 and SKOV3 cells. At least three independent experiments were performed.

The authors apologize for this error and state that this does not change the scientific conclusions of the article in any way. The original article has been updated.

